# Supplementary material for: Stromal PTEN determines mammary epithelial response to radiotherapy
Source: Nat Commun. 2018 Jul 17;9:2783. doi: 10.1038/s41467-018-05266-6 (PMC6050339; doi:10.1038/s41467-018-05266-6)
Supplement: Supplementary file 1 — Supplementary Information [file 41467_2018_5266_MOESM1_ESM.pdf]

SUPPLEMENTARY INFORMATION

Stromal PTEN determines mammary epithelial response to radiotherapy

Sizemore et al.

Supplementary Figures 1-11, Supplementary Tables 1-6

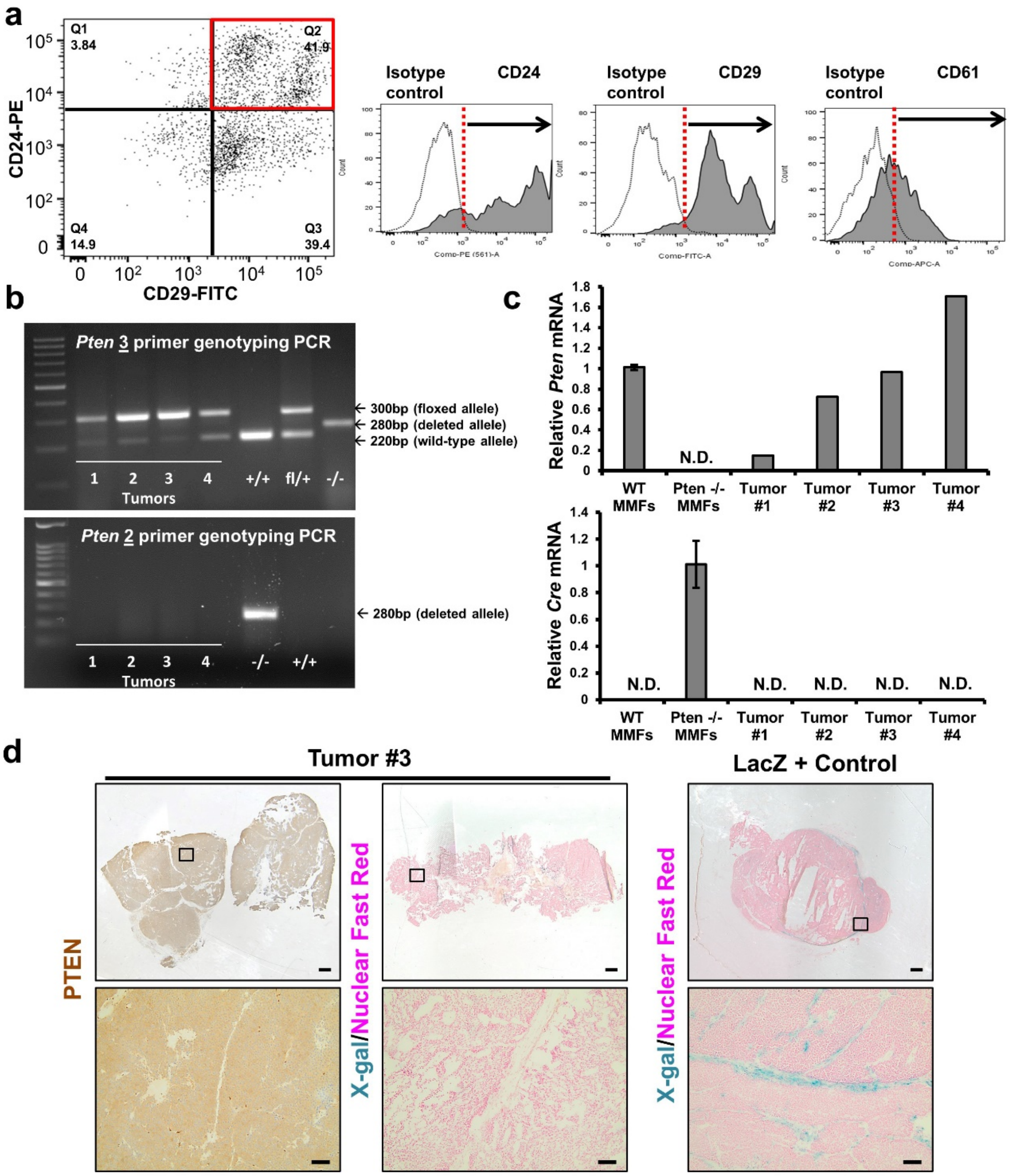

Supplementary Figure 1. Tumors arising from bulk CD24$^+$/CD29$^+$ *ErbB2;Fsp-cre;Pten$^{fl/fl}$* epithelium do not exhibit *Cre* recombinase expression and subsequent PTEN deletion

- **a)** Representative FACS plot defining CD24$^+$CD29$^+$ control mammary epithelium (*ErbB2;Pten$^{fl/fl}$*) and representative isotype control histograms exhibiting overlap between control and CD24-PE, CD29-FITC and CD61-APC positivity.
- **b)** Genotyping PCR for the wild-type (220bp), floxed (300bp) and deleted (280bp) *Pten* alleles on genomic DNA isolated from the four tumors that arose upon transplantation of CD24$^+$/CD29$^+$ bulk epithelium from *ErbB2;Fsp-cre;Pten$^{fl/fl}$* mice into wild-type syngeneic recipient mice (tumors depicted in Figure 4b,c). Control +/+ is wild-type, control fl/+ is *Pten$^{fl/+}$* and control -/- is *Fsp-cre;Pten$^{fl/fl}$* in the presence of *Fsp-cre* activity.
- **c)** *Pten* and *Cre* mRNA expression in wild-type control (WT = *Pten$^{fl/fl}$*) and PTEN-null (Pten -/- = *Fsp-cre;Pten$^{fl/fl}$*) MMFs, and in the four tumors that arose upon transplantation of CD24$^+$/CD29$^+$ bulk epithelium from *ErbB2;Fsp-cre;Pten$^{fl/fl}$* mice into wild-type syngeneic recipient mice (tumors depicted in Figure 4b,c). Bars represent mean expression of technical replicates relative to *Rpl4* ± s.e.m. N.D. = not detectable.
- **d)** (left) Representative PTEN immunostaining (brown) and X-gal/nuclear fast red staining of tumor #3 as depicted in **b** and **c**. (right) X-gal/nuclear fast red staining of an independent control frozen section processed for LacZ positivity at the same time as tumor #3. Top images – scale bar = 100mm; bottom images – scale bar = 100μm.

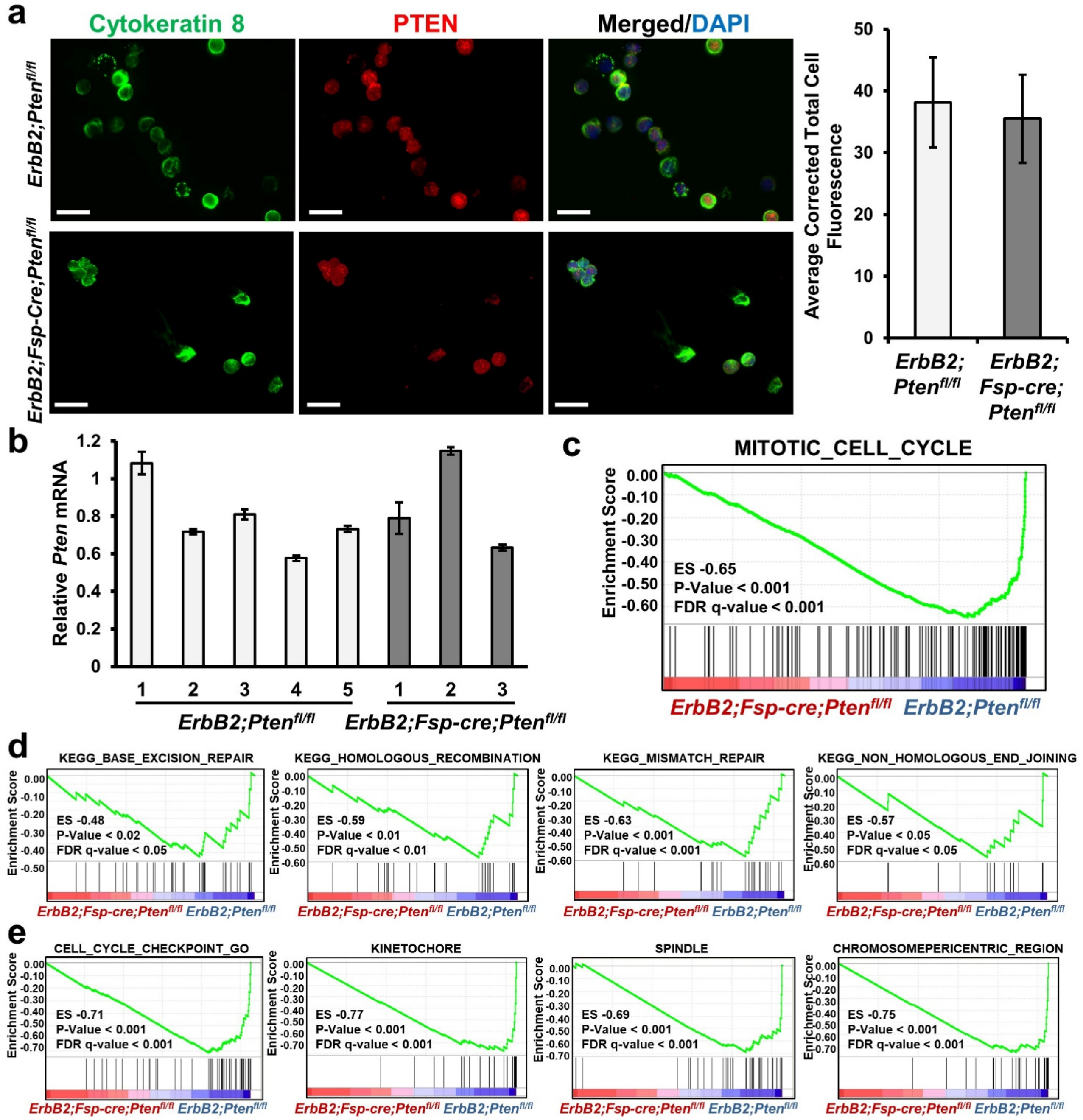

Supplementary Figure 2. Sorted CD24^{+}/CD29^{lo}/CD61^{-} *ErbB2;Fsp-cre;Pten$^{fl/fl}$* mature luminal epithelial cells exhibit a de-enrichment in multiple DNA repair and mitotic cell cycle gene sets without a reduction in PTEN expression

a) (left) PTEN immunofluorescence on cytospun mature luminal cells isolated from the mammary glands of *ErbB2;Pten$^{fl/fl}$* (n=3) and *ErbB2;Fsp-cre;Pten$^{fl/fl}$* (n=3) mice. Scale bar = 40µm. (right) Bars represent PTEN positivity as corrected total cell fluorescence ± s.e.m.

b) *Pten* mRNA expression in mature luminal cells: *ErbB2;Pten$^{fl/fl}$* (5 independent experiments of pooled mice) v. *ErbB2;Fsp-cre;Pten$^{fl/fl}$* (3 independent experiments of pooled mice). Bars represent mean expression of technical replicates relative to *Gapdh* ± s.e.m.

c) Gene set enrichment analysis for mitotic cell cycle genes in *ErbB2;Fsp-cre;Pten$^{fl/fl}$* versus control *ErbB2;Pten$^{fl/fl}$* mature luminal epithelium.

d) Gene set enrichment analysis for base excision repair, homologous recombination, mismatch repair, and non-homologous end-joining gene sets in *ErbB2;Fsp-cre;Pten$^{fl/fl}$* versus control *ErbB2;Pten$^{fl/fl}$* mature luminal epithelium.

e) Gene set enrichment analysis for cell cycle checkpoint, kinetochore, spindle, and chromosome/pericentric region gene sets in *ErbB2;Fsp-cre;Pten$^{fl/fl}$* versus control *ErbB2;Pten$^{fl/fl}$* mature luminal epithelium.

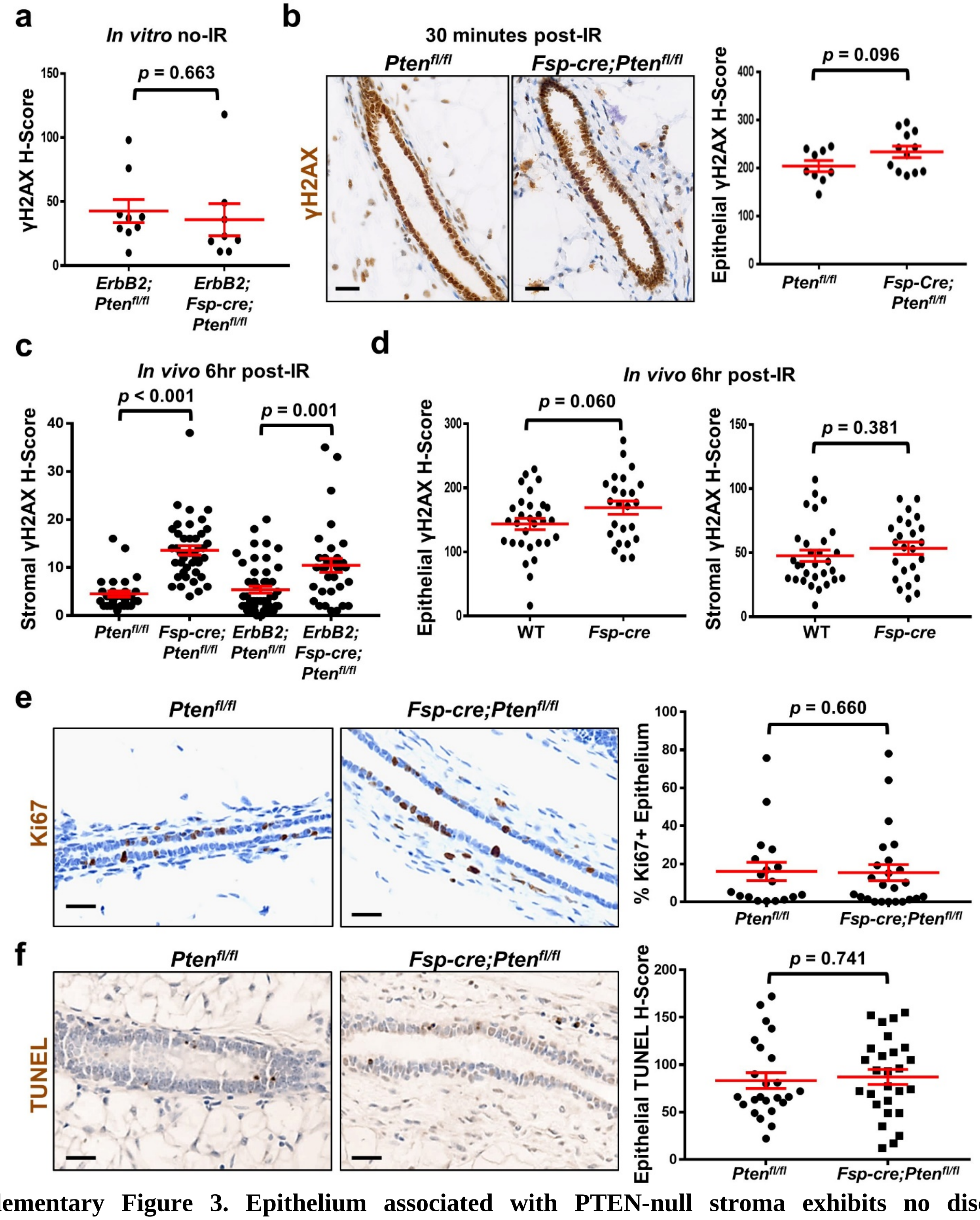

Supplementary Figure 3. Epithelium associated with PTEN-null stroma exhibits no discernable differences in baseline DNA damage, proliferation or apoptosis

a) Quantification (mean ± s.e.m.) of non-irradiated epithelial cells isolated from *ErbB2;Pten$^{fl/fl}$* and *ErbB2;Fsp-cre;Pten$^{fl/fl}$* mice cultured in parallel to the irradiated cells depicted in Figure 1f,g. *P* value determined by unpaired, two-tailed Student's *t* test (*ErbB2;Pten$^{fl/fl}$* n=9 fields/3 mice, *ErbB2;Fsp-cre;Pten$^{fl/fl}$* n=8 fields/3 mice).

b) Representative γ-H2AX immunohistochemistry and quantification (mean ± s.e.m.) of mammary epithelium in *Pten$^{fl/fl}$* and *Fsp-cre;Pten$^{fl/fl}$* mice irradiated (6 Gy whole-body) and evaluated 30 minutes post-radiation. *P* value determined by unpaired, two-tailed Student's *t* test (*Pten$^{fl/fl}$* n=9 fields/3 mice, *Fsp-cre;Pten$^{fl/fl}$* n=12 fields/4 mice). Scale bars = 20μm.

c) Quantification (mean ± s.e.m.) of mammary stroma in *Pten*$^{fl/fl}$, *Fsp-cre;Pten*$^{fl/fl}$, *ErbB2;Pten*$^{fl/fl}$ and *ErbB2;Fsp-cre;Pten*$^{fl/fl}$ mice irradiated (6 Gy whole-body) and evaluated 6 hours post-radiation. Both *p* values determined by two-tailed Mann-Whitney (*Pten*$^{fl/fl}$ n=30 fields/3 mice, *Fsp-cre;Pten*$^{fl/fl}$ n=40 fields/3 mice, *ErbB2;Pten*$^{fl/fl}$ n=49 fields/5 mice, *ErbB2;Fsp-cre;Pten*$^{fl/fl}$ n=33 fields/3 mice).

d) Quantification (mean ± s.e.m.) of mammary epithelial (left) and stroma (right) in wild-type and *Fsp-cre* mice irradiated (6 Gy whole-body) and evaluated 6 hours post-radiation. *P* values determined by unpaired, two-tailed Student's *t* test (wild-type n=30 fields/5 mice, *Fsp-cre* n=23 fields/4 mice).

e) Representative Ki67 quantification and immunohistochemistry of mammary epithelium in *Pten*$^{fl/fl}$ and *Fsp-cre;Pten*$^{fl/fl}$ mice irradiated (6 Gy whole-body) and evaluated 6 hours post-radiation. *P* value determined by two-tailed Mann-Whitney (*Pten*$^{fl/fl}$ n=18 fields/3 mice, *Fsp-cre;Pten*$^{fl/fl}$ n=24 fields/4 mice). Scale bars = 20µm.

f) Representative TUNEL quantification and immunohistochemistry of mammary epithelium in *Pten*$^{fl/fl}$ and *Fsp-cre;Pten*$^{fl/fl}$ mice irradiated (6 Gy whole-body) and evaluated 6 hours post-radiation. *P* value determined by unpaired, two-tailed Student's *t* test (*Pten*$^{fl/fl}$ n=24 fields/3 mice, *Fsp-cre;Pten*$^{fl/fl}$ n=27 fields/4 mice). Scale bars = 20µm.

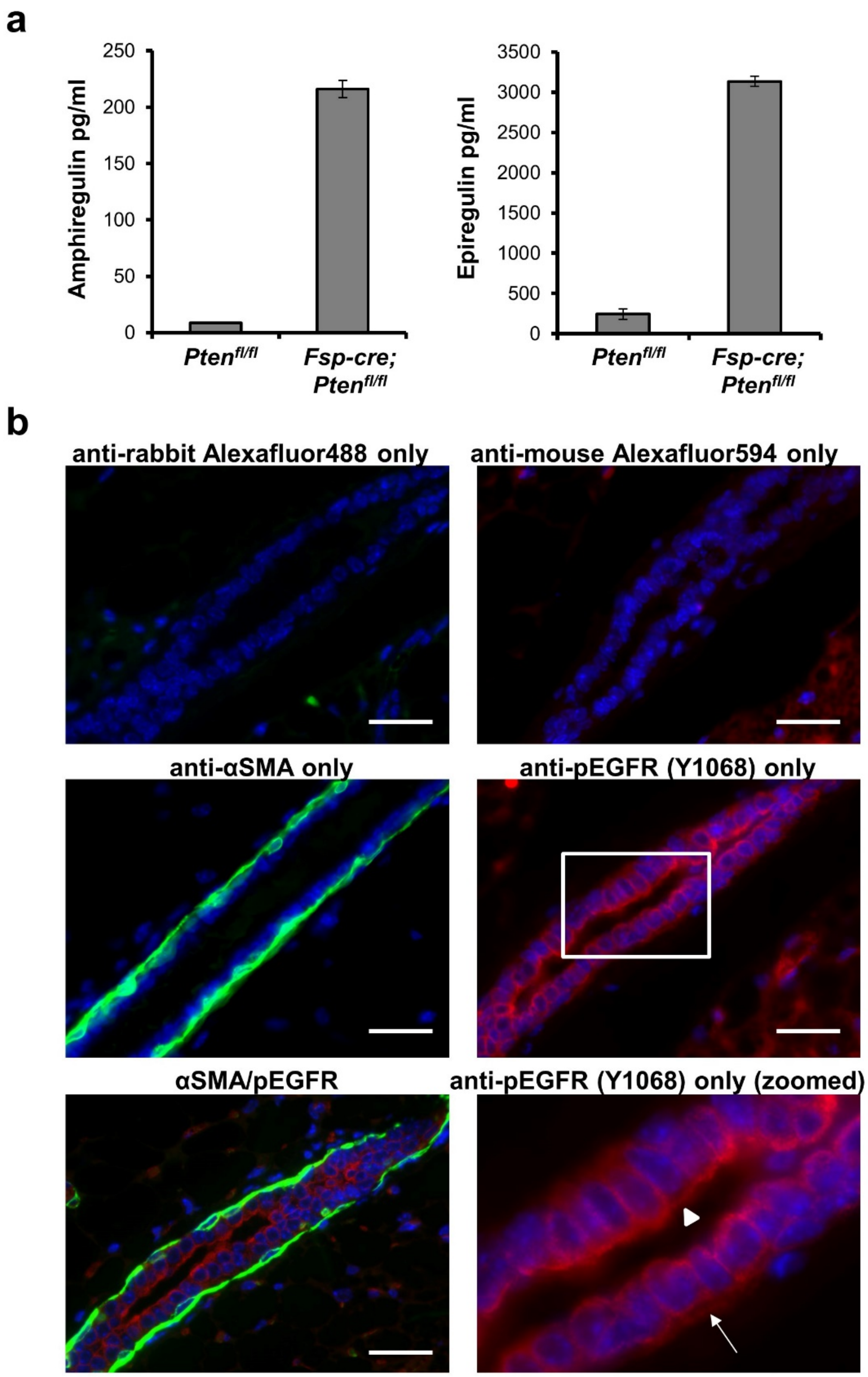

Supplementary Figure 4. PTEN-null MMFs secrete more amphiregulin and epiregulin protein versus wild-type control MMFs

a) Protein quantification by ELISA of amphiregulin (left) and epiregulin (right) levels in conditioned media isolated from immortalized control (*Pten*$^{fl/fl}$) and PTEN-null (*Fsp-cre;Pten*$^{fl/fl}$) MMFs. Bars represent mean expression of technical replicates ± s.e.m.

b) Representative phospho-EGFR (Y1068)/α-smooth muscle actin (αSMA) immunofluorescence in *ErbB2;Pten*$^{fl/fl}$ mammary epithelium. The arrow demarcates staining on the basolateral membrane. The arrowhead demarcates staining on the apical membrane. Scale bars = 25μm.

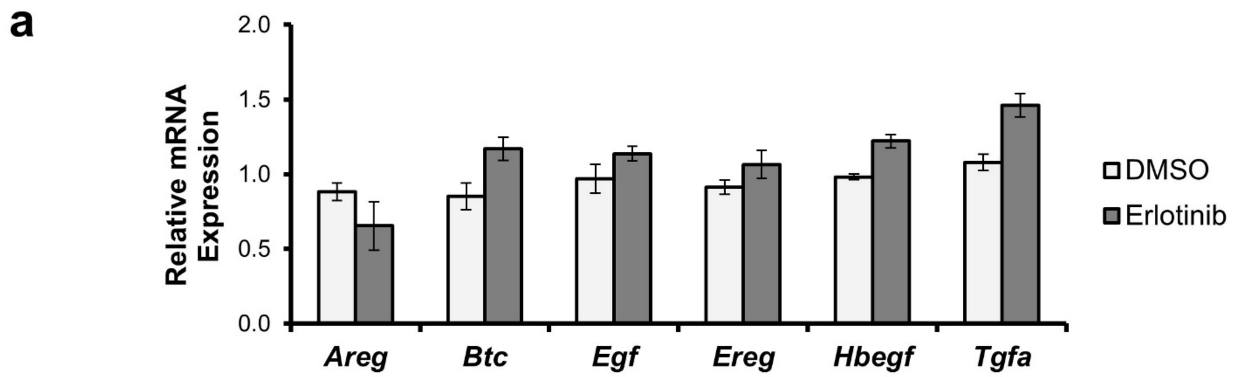

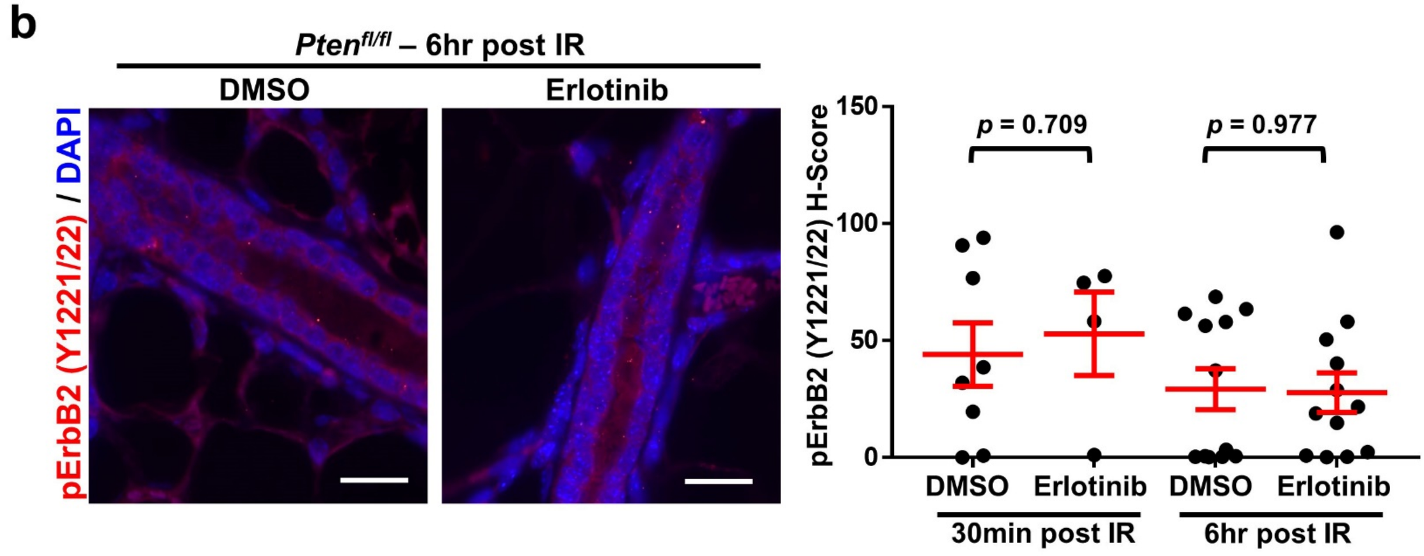

Supplementary Figure 5. Inhibition with the small molecule inhibitor erlotinib does not alter EGF ligand mRNA expression in PTEN-null MMFs *in vitro* and does not change ErbB2 epithelial activity *in vivo*

a) *Areg*, *Btc*, *Egf*, *Ereg*, *Hbegf* and *Tgfa* mRNA in immortalized PTEN-null (*Fsp-cre;Pten*$^{fl/fl}$) MMFs treated with and without 10µM erlotinib *in vitro*. Treatment was performed in DMEM containing 10% FBS and RNA isolated 24 hours post-treatment. Bars represent mean expression of technical replicates relative to *Gapdh* ± s.e.m.

b) Representative phospho-ErbB2 (Y1221/22) immunofluorescence and quantification (mean ± s.e.m.) of mammary epithelium in *Pten*$^{fl/fl}$ mice pre-treated with DMSO or erlotinib, irradiated (6 Gy whole-body) and evaluated 30 minutes or 6 hours post-radiation (images are 6 hour post IR). 30 minute time point *p* value determined by unpaired, two-tailed Student's *t* test (DMSO n=8 fields/2 mice, erlotinib n=4 fields/1 mouse). 6 hour time point *p* value determined by two-tailed Mann-Whitney (Vehicle n=12 fields/3 mice, erlotinib n=12 fields/3 mice). Scale bar = 20µm.

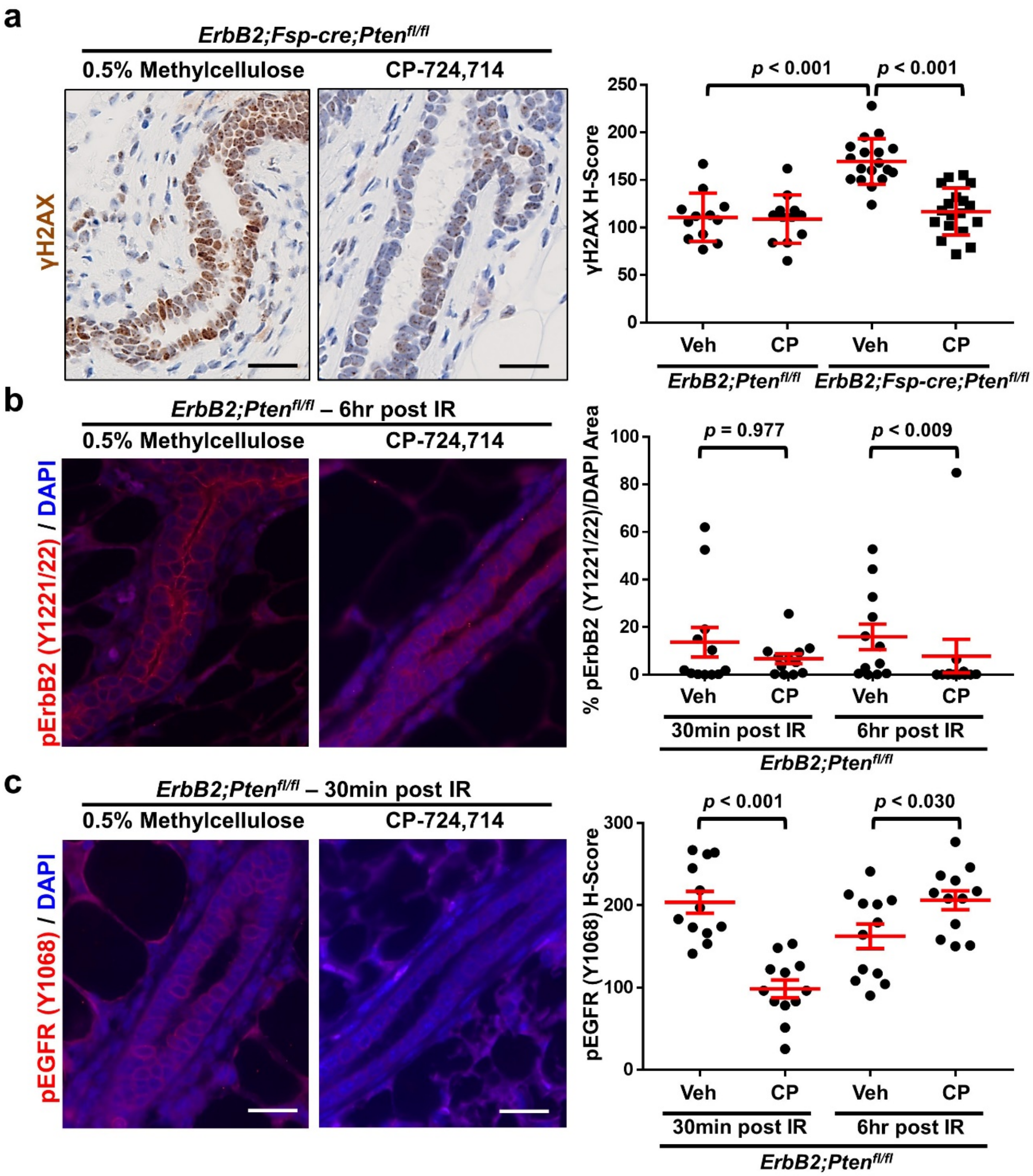

Supplementary Figure 6. Inhibition with the small molecule inhibitor CP-724,714 abrogates radiation-induced double strand DNA breaks in stromal PTEN-null associated epithelium

a) Representative γ-H2AX immunohistochemistry and quantification (mean ± s.e.m.) of mammary epithelium in *ErbB2;Pten$^{fl/fl}$* and *ErbB2;Fsp-cre;Pten$^{fl/fl}$* mice pre-treated with vehicle 0.5% Methylcellulose (Veh) or CP-724,714 (CP), irradiated (6 Gy whole-body) and evaluated 6 hours post-radiation. Both *p* values determined by unpaired, two-tailed Student's *t* test (*ErbB2;Pten$^{fl/fl}$*: veh n=12 fields/2 mice, CP n=12 fields/2 mice; *ErbB2;Fsp-cre;Pten$^{fl/fl}$*: veh n=18 fields/3 mice, CP n=18 fields/3 mice). Scale bar = 20μm.

b) Representative phospho-ErbB2 (Y1221/22) immunofluorescence and quantification (mean ± s.e.m.) of mammary epithelium in *ErbB2;Pten$^{fl/fl}$* mice pre-treated with 0.5% Methylcellulose (Veh) or CP-724,714 (CP), irradiated (6 Gy whole-body) and evaluated 30 minutes or 6 hours post-radiation (images are 6 hour post IR). Both *p* values determined by two-tailed Mann-Whitney (30 min: veh n=12 fields/2 mice, CP n=12 fields/2 mice; 6 hour: veh n=12 fields/2 mice, CP n=12 fields/2 mice). Scale bar = 20μm.

c) Representative phospho-EGFR (Y1068) immunofluorescence and quantification (mean ± s.e.m.) of mammary epithelium in *ErbB2;Pten$^{fl/fl}$* mice pre-treated with 0.5% Methylcellulose (Veh) or CP-724,714 (CP), irradiated (6 Gy whole-body) and evaluated 30 minutes or 6 hours post-radiation (images are 30 minutes post IR). Both *p* values determined by unpaired, two-tailed Student's *t* test (30 min: veh n=12 fields/2 mice, CP n=12 fields/2 mice; 6 hourr: veh n=12 fields/2 mice, CP n=12 fields/2 mice). Scale bar = 20μm.

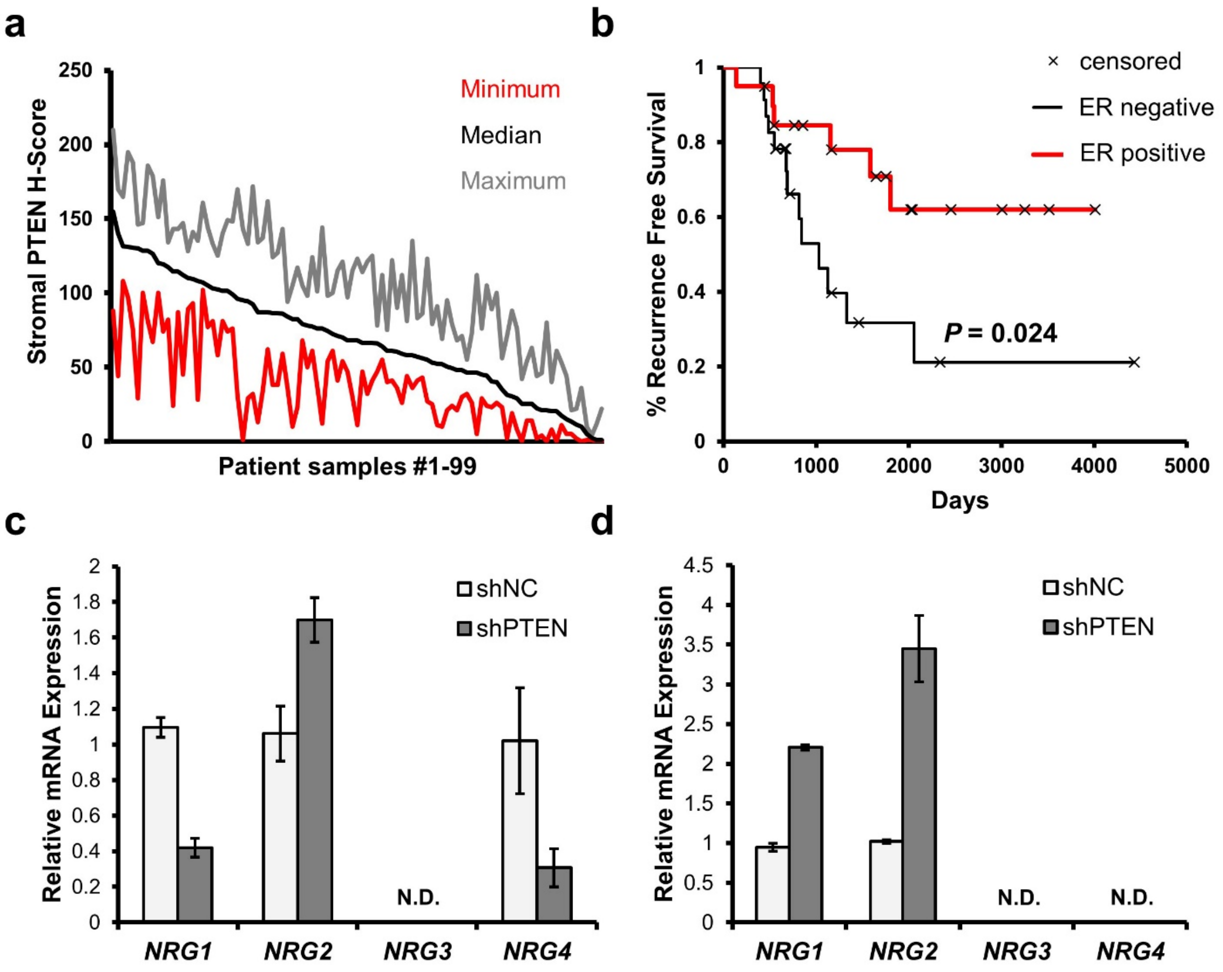

Supplementary Figure 7. Estrogen Receptor (ER) status predicts outcome within the OSUCCC HER2-positive patient cohort

a) Quantification (4-10 fields per sample) in normal breast tissue isolated from women who underwent reduction mammoplasty (x-axis shows all samples, n=99). Maximum H-score field per sample indicated by the gray line, median H-score per sample indicated by the gray line and minimum H-score field indicated by the red line.

b) Kaplan-Meier analysis exhibiting recurrence probability within the HER2-positive patient population stratified by ER status (ER-positive, n=20; ER-negative, n=23). *P* value determined by Log-rank (Cox-Mantel).

c) Neuregulin ligand mRNA expression in cancer associated fibroblasts isolated from a breast cancer patient with and without PTEN lentiviral knockdown (same sample as Figure 5e). Bars represent mean expression of technical replicates relative to *Gapdh* ± s.e.m. *NRG3* is not detectable (N.D.).

d) Neuregulin ligand mRNA expression in normal human breast fibroblasts (>10cm from tumor) with and without PTEN lentiviral knockdown (same sample as Figure 5f). Bars represent mean expression of technical replicates relative to *Gapdh* ± s.e.m. *NRG3* and *NRG4* are not detectable (N.D.).

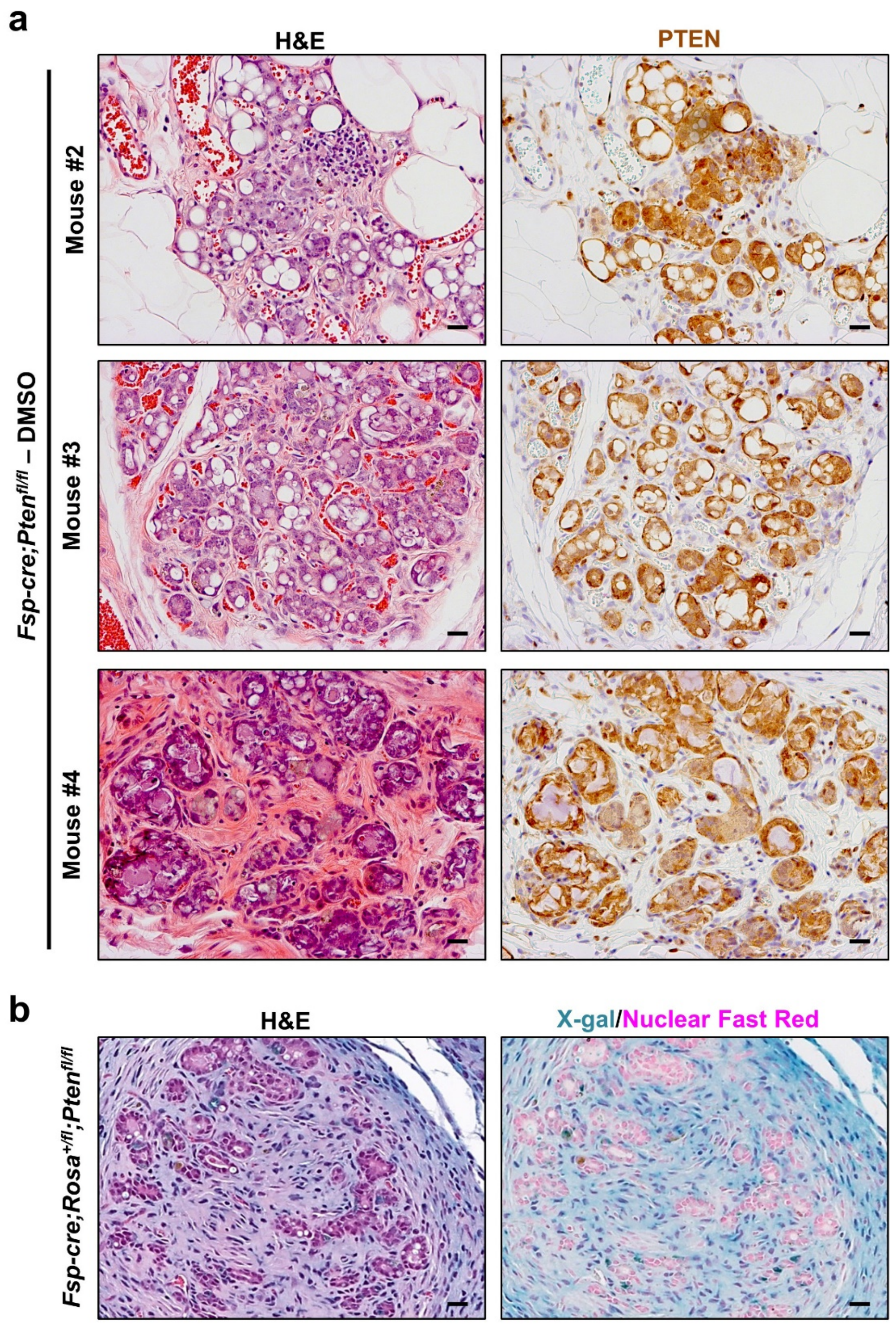

Supplementary Figure 8. ***Fsp-cre*** **activity is specific to the mammary stroma with hyperplastic lesions maintaining PTEN positivity**

a) PTEN immunohistochemistry and associated H&E in *Fsp-cre;Pten*$^{fl/fl}$ mammary tissue in three additional mice pre-treated with DMSO, irradiated (6 Gy whole-body), transplanted and evaluated 10 months post radiation. All three tissues display moderate lobuloalveolar hyperplasia. Scale bar = 20μm.

b) Representative H&E and X-gal/nuclear fast red staining of *Fsp-cre;Rosa*$^{+/fl}$*;Pten*$^{fl/fl}$ mammary tissue in a mouse pre-treated with erlotinib, irradiated (6 Gy whole-body), transplanted and evaluated ~10 months post radiation. Scale bar = 20μm.

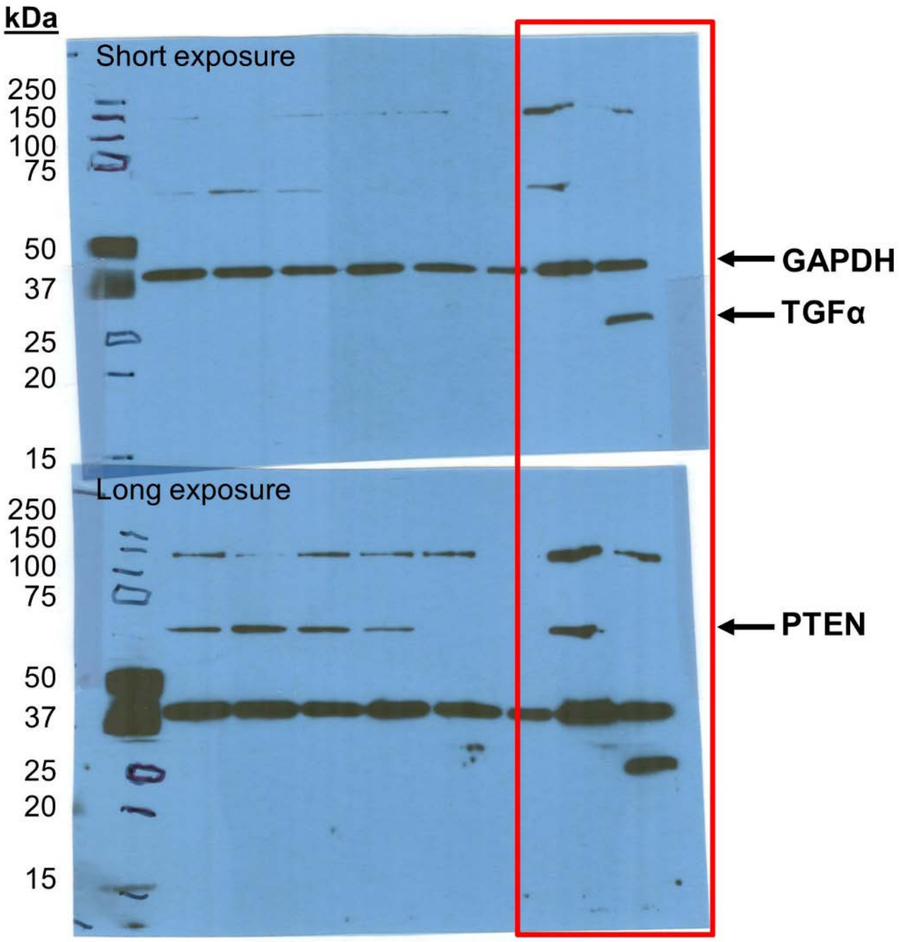

Supplementary Figure 9. Uncropped images from western blot in Figure 3b.

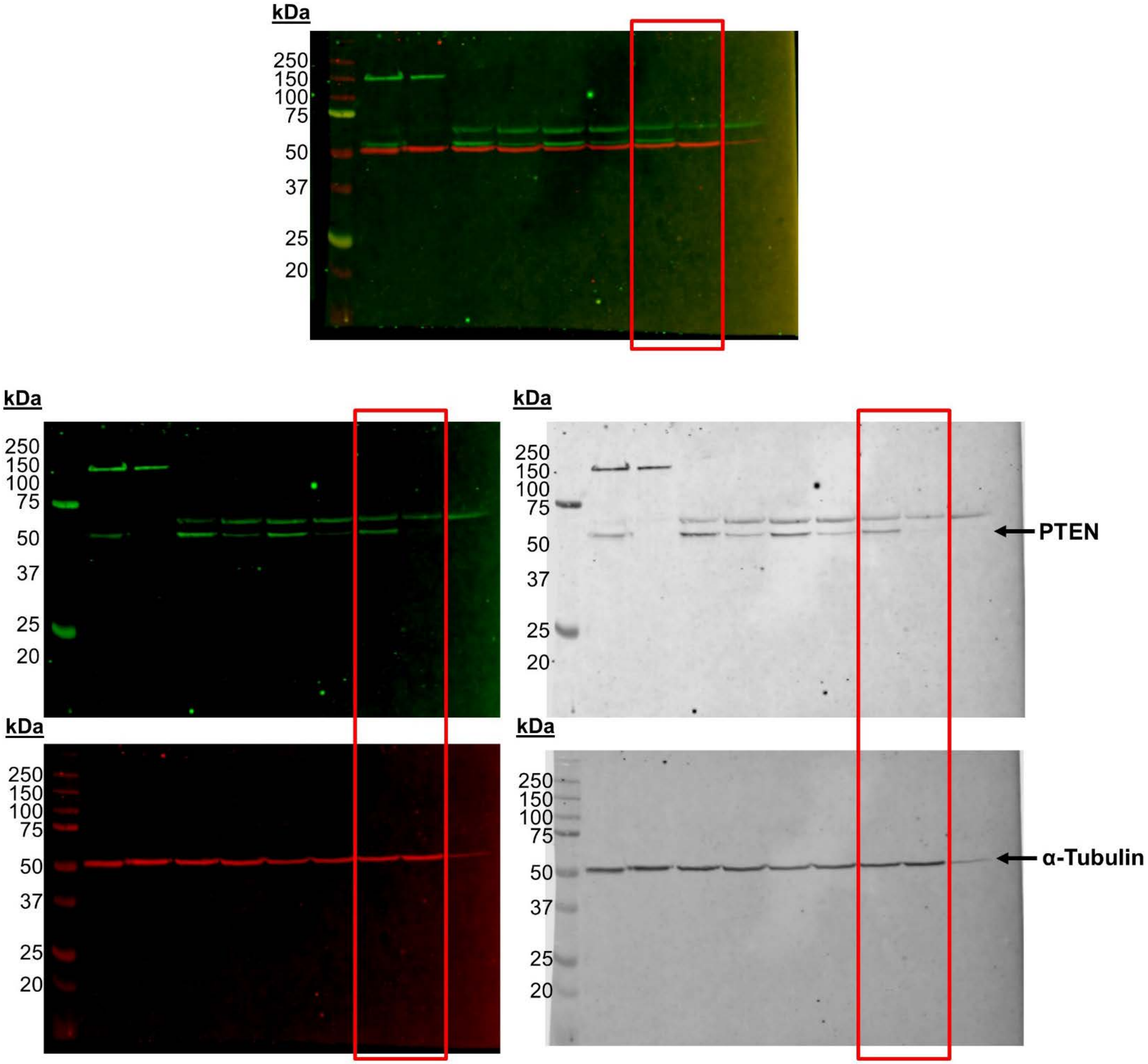

Supplementary Figure 10. Uncropped images from western blot in Figure 5e.

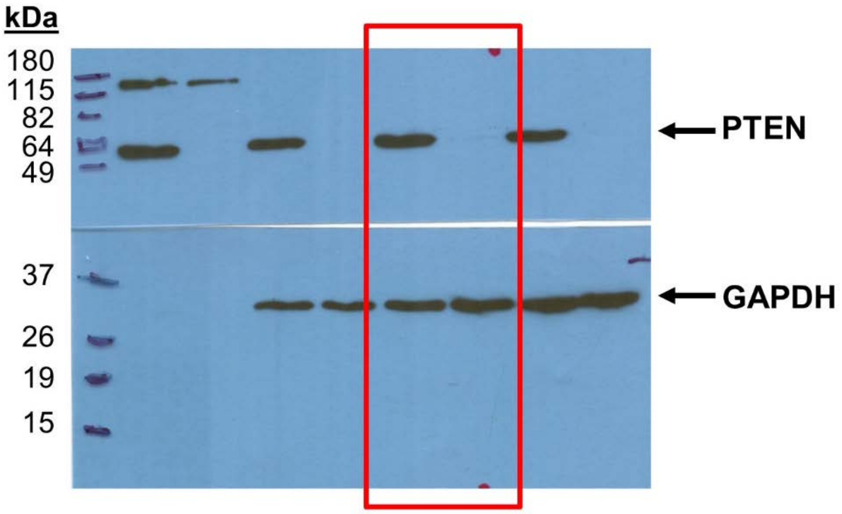

Supplementary Figure 11. Uncropped image from western blot in Figure 5f.

NAME	SIZE	ES	NES	NOM p-val	FDR q-val	FWER p-val	RANK AT MAX	LEADING EDGE
**MITOTIC_CELL_CYCLE**	**136**	**-0.64572**	**-2.95393**	**0**	**0**	**0**	**2112**	**tags=51%, list=16%, signal=60%**
CELL_CYCLE_PROCESS	171	-0.61372	-2.87485	0	0	0	2234	tags=49%, list=17%, signal=58%
MITOSIS	74	-0.6925	-2.86416	0	0	0	1870	tags=54%, list=14%, signal=63%
M_PHASE_OF_MITOTIC_CELL_CYCLE	76	-0.69365	-2.85649	0	0	0	1870	tags=54%, list=14%, signal=63%
M_PHASE	100	-0.63886	-2.79309	0	0	0	2112	tags=51%, list=16%, signal=60%
CELL_CYCLE_PHASE	152	-0.60176	-2.77323	0	0	0	2112	tags=46%, list=16%, signal=54%
CHROMOSOMAL_PART	86	-0.6402	-2.73929	0	0	0	2264	tags=51%, list=17%, signal=61%
CHROMOSOME	109	-0.61612	-2.72727	0	0	0	2264	tags=49%, list=17%, signal=58%
CELL_CYCLE_GO_0007049	275	-0.54269	-2.71105	0	0	0	2234	tags=40%, list=17%, signal=48%
MICROTUBULE_CYTOSKELETON	131	-0.57753	-2.63179	0	0	0	2355	tags=49%, list=18%, signal=59%
**CELL_CYCLE_CHECKPOINT_GO_0000075**	**44**	**-0.71286**	**-2.60189**	**0**	**0**	**0**	**2685**	**tags=66%, list=20%, signal=83%**
REGULATION_OF_CELL_CYCLE	158	-0.54949	-2.54367	0	0	0	2825	tags=47%, list=21%, signal=59%
REGULATION_OF_MITOSIS	39	-0.71721	-2.5341	0	0	0	2112	tags=59%, list=16%, signal=70%
GOLGI_APPARATUS_PART	82	-0.59255	-2.49568	0	0	0	2609	tags=49%, list=20%, signal=60%
**SPINDLE**	**36**	**-0.68822**	**-2.47256**	**0**	**0**	**0**	**2280**	**tags=64%, list=17%, signal=77%**
**CHROMOSOMEPERICENTRIC_REGION**	**26**	**-0.75183**	**-2.43462**	**0**	**0**	**0**	**2004**	**tags=65%, list=15%, signal=77%**
**KINETOCHORE**	**22**	**-0.77446**	**-2.42137**	**0**	**0**	**0**	**791**	**tags=50%, list=6%, signal=53%**
GOLGI_VESICLE_TRANSPORT	45	-0.64324	-2.38827	0	0	0	3580	tags=73%, list=27%, signal=100%
CHROMOSOME_SEGREGATION	26	-0.72315	-2.37478	0	0	0	2264	tags=65%, list=17%, signal=79%
**DNA_REPAIR**	**115**	**-0.52128**	**-2.34313**	**0**	**0**	**0**	**4183**	**tags=60%, list=32%, signal=87%**
INTRACELLULAR_TRANSPORT	248	-0.47417	-2.34244	0	0	0	3949	tags=58%, list=30%, signal=81%
GOLGI_APPARATUS	189	-0.49236	-2.33625	0	0	0	2687	tags=41%, list=20%, signal=51%
NUCLEAR_PORE	26	-0.69794	-2.31698	0	0	0	2695	tags=69%, list=21%, signal=87%

Supplementary Table 1. C2 curated datasets de-enriched in the *ErbB2;Fsp-cre;Pten*$^{fl/fl}$ mature luminal epithelium versus *ErbB2;Pten*$^{fl/fl}$ epithelium

Criteria	Total population		PTEN high		PTEN low	
	Number	%	Number	%	Number	%
**Age at diagnosis**						
<50	5	12	2	5	3	7
>50	38	88	20	47	18	42
**Grade**						
I	3	7	2	5	1	2
II	8	19	3	7	5	12
III	30	70	16	37	14	33
Not determined	2	5	1	2	1	2
**AJCC Stage**						
1, 1A	13	30	3	7	10	23
2A, 2B	16	37	8	19	8	19
3A, 3B, 3C	14	33	11	26	3	7
**Estrogen Receptor**						
Positive	20	47	7	16	13	30
Negative	23	53	15	35	8	19
**Progesterone Receptor**						
Positive	18	42	8	19	10	23
Negative	25	58	14	33	11	26
**1st Course RT**						
Breast	25	58	11	26	14	33
Breast/Lymph Nodes	3	7	2	5	1	2
Chest Wall	6	14	5	12	1	2
Chest Wall/Lymph Nodes	5	12	3	7	2	5
Lymph nodes, NOS	2	5	1	2	1	2
Unknown	2	5	0	0	2	5
**PTEN**						
High	22	51				
Low	21	49				

Supplementary Table 2. Clinicopathological characteristics of HER2-positive patient population treated with radiation (N=43)

Genotype	Radiation	Drug	Lobule type	Lobule 2-3 distribution	Lobulaveolar hyperplasia	Other alveolar/ductal lesions	Score
Pten$^{fl/fl}$	No	none	1				0
Pten$^{fl/fl}$	No	none	1				0
Pten$^{fl/fl}$	No	none	1				0
Pten$^{fl/fl}$	No	none	1				0
Pten$^{fl/fl}$	Yes	DMSO	1				0
Pten$^{fl/fl}$	Yes	DMSO	1				0
Pten$^{fl/fl}$	Yes	DMSO	1				0
Pten$^{fl/fl}$	Yes	DMSO	1				0
Pten$^{fl/fl}$	Yes	DMSO	1				0
Pten$^{fl/fl}$	Yes	DMSO	0				0
Pten$^{fl/fl}$	Yes	DMSO	2	Focal	Minimal		1
Pten$^{fl/fl}$	Yes	DMSO	1				0
Pten$^{fl/fl}$	Yes	DMSO	1				0
Pten$^{fl/fl}$	Yes	Erlotinib	1				0
Pten$^{fl/fl}$	Yes	Erlotinib	1				0
Pten$^{fl/fl}$	Yes	Erlotinib	1				0
Pten$^{fl/fl}$	Yes	Erlotinib	1				0
Pten$^{fl/fl}$	Yes	Erlotinib	1				0
Pten$^{fl/fl}$	Yes	Erlotinib	1				0
Pten$^{fl/fl}$	Yes	Erlotinib	1				0
Pten$^{fl/fl}$	Yes	Erlotinib	1				0
Pten$^{fl/fl}$	Yes	Erlotinib	1				0
Fsp-cre;Pten$^{fl/fl}$	No	none	1				0
Fsp-cre;Pten$^{fl/fl}$	No	none	1				0
Fsp-cre;Pten$^{fl/fl}$	No	none	2	Multifocal	Mild		2
Fsp-cre;Pten$^{fl/fl}$	No	none	1				0
Fsp-cre;Pten$^{fl/fl}$	Yes	DMSO	2	Multifocal	Mild		2
Fsp-cre;Pten$^{fl/fl}$	Yes	DMSO	3	Multifocal to coalescing	Moderate		3
Fsp-cre;Pten$^{fl/fl}$	Yes	DMSO	3	Multifocal to coalescing	Moderate		3
Fsp-cre;Pten$^{fl/fl}$	Yes	DMSO	2	Multifocal	Mild	Focal hyperplasia with atypia	4
Fsp-cre;Pten$^{fl/fl}$	Yes	DMSO	3	Multifocal to coalescing	Moderate		3
Fsp-cre;Pten$^{fl/fl}$	Yes	DMSO	3	Multifocal to coalescing	Moderate		3
Fsp-cre;Pten$^{fl/fl}$	Yes	DMSO	3	Multifocal to coalescing	Moderate		3
Fsp-cre;Pten$^{fl/fl}$	Yes	DMSO	1				0
Fsp-cre;Pten$^{fl/fl}$	Yes	DMSO	2	Multifocal	Mild		2
Fsp-cre;Pten$^{fl/fl}$	Yes	Erlotinib	2	Focal	Minimal		1
Fsp-cre;Pten$^{fl/fl}$	Yes	Erlotinib	1				0
Fsp-cre;Pten$^{fl/fl}$	Yes	Erlotinib	2	Multifocal	Mild		2
Fsp-cre;Pten$^{fl/fl}$	Yes	Erlotinib	2	Focal	Minimal		1
Fsp-cre;Pten$^{fl/fl}$	Yes	Erlotinib	1				0
Fsp-cre;Pten$^{fl/fl}$	Yes	Erlotinib	2	Focal	Minimal		1
Fsp-cre;Pten$^{fl/fl}$	Yes	Erlotinib	1				0
Fsp-cre;Pten$^{fl/fl}$	Yes	Erlotinib	1				0

Supplementary Table 3. Phological overview of transplantation cohort

Gene	Sense	Sequence	Product size
MMTV-Neu	Forward	GGAACCTTACTTCTGTGGTGTGAC	500bp
MMTV-Neu	Reverse	TAGCAGACACTCTATGCCTGTGTG	
Pten^{WT}	Common forward	GGGTTACACTAACTAAACGAGTCC	220bp
Pten^{loxP}	loxP reverse	GAATGCCATTACCTAGTAAAGCAAGG	300bp
Pten$^{-/-}$	Deleted reverse	GAATGATAATAGTACCTACTTCAG	280bp

Supplementary Table 4. Genotyping primer sequences

Gene	Forward	Reverse	UPL Probe
Cre	gttttgccgggtcagaaaa	tcaatcgatgagttgcttcaa	#20
Pten	aggcacaagaggccctagat	ctgactgggaattgtgactcc	#60
Rpl4	gatgagctgtatggcacttgg	cttgtgcatgggcaggtta	#38

Supplementary Table 5. Roche Universal Probe Library (UPL) qRT-PCR primer sequences and probe numbers.

Gene	Mouse ID	Human ID
AREG	Mm00437583_m1	Hs00950669_m1
BTC	Mm00432137_m1	Hs01101204_m1
EGF	Mm00438696_m1	Hs01099999_m1
EREG	Mm00514794_m1	Hs00914313_m1
HBEGF	Mm00439306_m1	Hs00181813_m1
NRG1	Mm01212130_m1	Hs00247620_m1
NRG2	Mm01158087_m1	Hs00171706_m1
NRG3	Mm01209104_m1	Hs01377907_m1
NRG4	Mm00446254_m1	Hs00945535_m1
Pten	Mm00477208_m1	
TGFA	Mm00446232_m1	Hs00608187_m1
GAPDH	Mm99999915_g1	Hs99999905_m1

Supplementary Table 6. Applied Biosystems TaqMan® Gene Expression Assay IDs.
